# Amaranth and buckwheat grains: Nutritional profile, development of functional foods, their pre-clinical cum clinical aspects and enrichment in feed

**DOI:** 10.1016/j.crfs.2024.100836

**Published:** 2024-09-02

**Authors:** Harsh Kumar, Shivani Guleria, Neetika Kimta, Rajni Dhalaria, Eugenie Nepovimova, Daljeet Singh Dhanjal, Suliman Y. Alomar, Kamil Kuca

**Affiliations:** a Centre of Advanced Technologies, Faculty of Science, University of Hradec Kralove, Rokitanskeho 62, 50003, Hradec Kralove, Czech Republic; b Department of Biotechnology, TIFAC-Centre of Relevance and Excellence in Agro and Industrial Biotechnology (CORE), Thapar Institute of Engineering and Technology, Patiala, 147001, India; c School of Biological and Environmental Sciences, Shoolini University of Biotechnology and Management Sciences, Solan, 173229, India; d Department of Chemistry, Faculty of Science, University of Hradec Kralove, 50003, Hradec Kralove, Czech Republic; e School of Bioengineering and Biosciences, Lovely Professional University, Phagwara, 144411, India; f Zoology Department, College of Science, King Saud University, Riyadh, 11451, Saudi Arabia; g Biomedical Research Center, University Hospital of Hradec Kralove, 50005, Hradec Kralove, Czech Republic

**Keywords:** Bioactive compounds, Food insecurity, Health benefits, Product development, Pseudocereal grains

## Abstract

The resurgence of interest in amaranth and buckwheat as nutrient-rich and versatile grains has incited extensive research aimed at exploring their potential benefits for sustainable agriculture and human nutrition. Amaranth is renowned for its gluten-free nature and exceptional nutritional profile, offering high-quality proteins, fiber, minerals, and bioactive compounds. Similarly, buckwheat is recognized for its functional and nutraceutical properties, offering a plethora of health benefits attributed to its diverse array of biologically active constituents; flavonoids, phytosterols, and antioxidants. This comprehensive review comprehends the existing understanding of the composition, anti-nutritional factors, biological activity, and potential application of these grains, emphasizing their pivotal role in addressing global food insecurity. Developed functional foods using these grains are having enhanced physicochemical properties, mineral content, phenolic content and overall sensory acceptability. In addition, the consumption of developed functional food products proved their health benefits against various type of anomalies. Moreover, enrichment of both grains in the animal feeds also showing positive health benefits.

## Highlights

• Amaranth and buckwheat are considered underutilized grains but have a rich nutritional profile.
• Both grains are now popular for the production of gluten-free bakery products.
• Functional food designed using both grains clinically proven their biological effects.
• Feeding of these grains increases the body mass, egg production, crude protein and fat in the livestock.

## Graphical abstract

**Figure:**
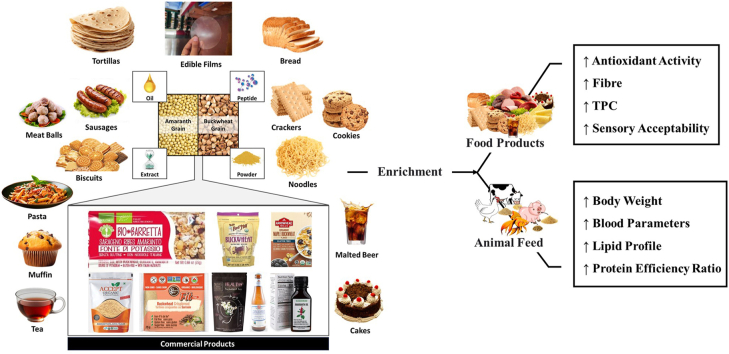

## 1 Introduction

The United Nations Organization (UNO) predicts that by 2030, more than 600 million individuals will experience hunger on a global scale, highlighting the immense challenge of achieving the objective of eliminating hunger entirely. The United Nations research indicates that the world is deviating from its objective of eradicating malnutrition, food insecurity, and hunger in all forms by 2030 (Kumar et al., 2023a). In order to achieve food security, it demands a comprehensive approach that involves the incorporation of social protection measures to guarantee access to safe and nutritious food, especially for children, and transforming food systems to promote a more inclusive and sustainable global environment (UNO, 2023). Allocating funds to both rural and urban areas, as well as implementing social protection measures, is crucial in order to guarantee that those living in poverty have adequate access to food and may improve their overall quality of life (UNO, 2023). Functional Food Science in Europe (FUFOSE) describes functional food as a kind of food that not only has nutritional value but also possesses at least one advantageous impact on the human body. Overall, there is an expectation of reducing the probability of diseases (Kumar et al., 2023b). A nutritious meal, whether composed of natural ingredients or fortified with additional substances, has the capacity to improve the efficiency and output of persons across various age groups or specific age brackets (Jiménez-Colmenero et al., 2018; Ashaolu, 2020; Das et al., 2020; Gong et al., 2020). Research conducted by Tripathy et al. (2021) has highlighted that the inclusion of plant components in functional diets can significantly improve human health.

The grain functionality primarily relies on the genetic composition and the influence of environmental conditions on its key constituents, such as carbs, proteins, vitamins, minerals, and phenolic phytochemicals (Mir et al., 2018). As a result, many cereal crops may have an abundance of one component while lacking another molecule. To address this problem, significant focus has been directed towards the exploration and application of unconventional food crops, such as pseudocereals (Mir et al., 2018). Pseudocereals (amaranth, quinoa, and buckwheat) have garnered significant attention owing to their high nutritional value, phytochemical content, and use in the production of gluten-free products (Mir et al., 2018). This novel botanical species plays a pivotal part in the advancement and proliferation of agricultural commodities and sustenance. Consequently, developing new food products from plant species that have numerous health advantages has a strong potential to improve public health. Consequently, these cuisines are progressively gaining more attention from scientists, consumers, and food producers (Gul et al., 2016). Despite their nutritional richness in minerals, amino acids, protein, and other compounds, these products have limited commercialization due to the absence of modern technology, equipment, and research advancements (Rao and Poonia, 2023).

Prebiotics, phytogenic preparations, and direct-fed microorganisms (DFM) are examples of functional feed additives that have improved animal health, growth performance, and microbial food safety in chickens. These additives have also been regarded as potential components of antibiotic-free animal production (Broderick et al., 2021). The conversion of food into biomass gain within the digestive system of animals directly affects the condition and well-being of the animals, which subsequently has a significant impact on the farmers financial performance (Kumar et al., 2023b). The demand for raw resources, such as maize, is substantially high due to its significant role in human diets. Owing to this, the demand for raw resources utilized in animal feed is steadily rising. Hence, it is imperative to explore other sources of energy and protein (Aderibigbe et al., 2022). The current review aims to elucidate the nutritional profile as well as the animal and human studies on grain flour proteins and peptides of amaranth and buckwheat. It also discusses the anti-nutritional aspects of these two grains along with technologies used for their removal. Additionally, the application of these two grains in food enrichment in cereal-based foods, meat-based foods, beverage enrichment, and films/coating has also been discussed. Furthermore, pre-clinical and clinical studies investigating the effect of food products developed using these two grains have also been evaluated. Finally, the enrichment of animal feeds with these two grains have been comprehended to understand the potential health benefits on their consumption.

## 2 Nutritional profile

**Fig. 1:**
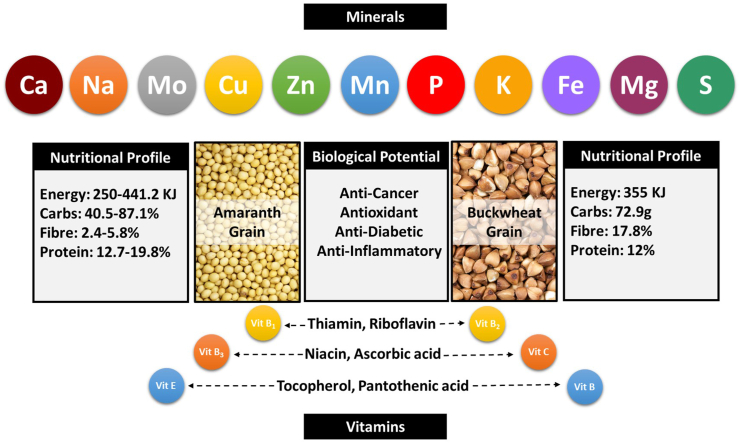
Amaranth and buckwheat grains: nutritional profile and biological activities.

Amaranth and buckwheat are rich sources of fatty acids, polyphenols, vitamins, minerals, nutrients, and amino acids (Table 1). An analysis of the effects of amaranth grains on inflammation, obesity, diabetes, and cardiovascular disease was done by Tang and Tsao (2017). They found that these effects were primarily due to the presence of flavonoids and phenolic acids, as shown in Fig. 1. Grundy et al. (2020) examined the speed and degree of fat breakdown in 14 types of amaranth grains (in the form of flour) obtained from various sources found in the markets of Kenya. It was postulated that the physicochemical features of amaranth grains' cell walls are influenced by their origin (genotype and/or growth circumstances) and behaviour during processing and digestion in the human gastrointestinal system. Further, their research unveiled that the cell wall of amaranth serves as a barrier that obstructs the transport of enzymes and other chemicals essential for digestion. Montoya-Rodríguez et al. (2015), in their review, outlined the approximately fifteen significant proteins found in amaranth seeds. The proteins mentioned are 11S globulin, 7S globulin, α-amylase inhibitor, acetolactate synthase, amaranth albumin 1, antimicrobial proteins, glucose-1-phosphate adenyl transferase, glucosyltransferase, granule-bound starch synthase 1, non-specific lipid-transfer-protein-1, polyamine oxidase, prosystemin, ring-zinc finger protein, superoxide dismutase and trypsin inhibitor. The proteins showed a notable presence of angiotensin-converting enzyme-inhibitor peptides, as well as dipeptidyl peptidase IV inhibitor. Additionally, certain proteins were found to possess antioxidant, glucose uptake-enhancing, antithrombotic and anticancer properties. Popoola (2022) discovered that Nigerian *Amaranthus viridis* seeds contain a significant amount of phenolic chemicals including ferulic acid and para-hydroxybenzoic acid. The range of total dietary fiber content in 21 genotypes of *Amaranthus cruentus* was between 10 % and 25 % (Mustafa et al., 2011). Zhu (2020) found that all amaranth species contained a higher amount of insoluble fiber compared to soluble fiber.

**Table 1:** Nutritional value of amaranth and buckwheat grains.

Amaranth nutrients	Value per 100 gm	Buckwheat nutrients	Value per 100 gm
Energy (KJ)	250.8–441.2	Energy (KJ)	355
Moisture (%)	6.5–11.1	Moisture (%)	11
Ash (%)	2.2–3.5	Ash	–
Fat (%)	1.7–10.3	Fat (g)	7.4
Carbohydrate (%)	40.5–87.1	Carbohydrate (g)	72.9
Crude fibre (%)	2.4–5.8	Crude fibre (%)	17.8
Protein (%)	12.7–19.8	Crude protein (%)	12
Starch (%)	49.5–73	Starch	–
Amino acids (g)		Amino acids (μmoles/g)	
Tryptophan	1.3	Tryptophan	32.69
Methionine	4.4	Methionine	21.54
Cysteine	4.4	Cysteine	59.7
Threonine	2.9	Threonine	64.74
Isoleucine	3	Isoleucine	45.1
Valine	3.6	Valine	68.07
Lysine	5	Lysine	51.13
Phenylalanine	6.4	Phenylalanine	64.37
Tyrosine	6.4	Tyrosine	32.39
Leucine	4.7	Leucine	89.42
Minerals (mg/kg)		Minerals (mg/kg)	
Calcium (Ca)	1463–2000	Calcium (Ca)	117.52
Magnesium (Mg)	2466.2–3280	Magnesium (Mg)	2573.5
Iron (Fe)	65.4–660	Iron (Fe)	24.81
Potassium (K)	4005–5520	Potassium (K)	4978.26
Phosphorous (P)	4731.25–6630	Phosphorous (P)	4384.1
Sulphur (S)	2072.50	Manganese (Mn)	13.69
Manganese (Mn)	8.8–57.1	Zinc (Zn)	25.32
Zinc (Zn)	28.9–113	Copper (Cu)	5.6
Copper (Cu)	2.8–10.7	Molybdenum (Mo)	0.41
		Sodium (Na)	14.36
Vitamins (mg/kg)		Vitamins (mg/100g)	
Thiamin	0.3–0.9	Thiamin	3.3
Riboflavin	0.1–0.7	Riboflavin	10.6
Niacin	1.042	Niacin	18.0
Ascorbic acid	30	Tocopherol	40
		Pantothenic acid	11
		Choline	440
Polyphenols (mg/kg)		Polyphenols (mg/kg)	
Vanillic acid	15.5–69.5	Vanillic acid	–
Gallic acid	11.0–440	Gallic acid	–
*p*-Coumaric acid	1.2–17.4	Trans-coumaric acid	110
Ferulic acid	120–620.0	Trans-ferulic acid	4
Caffeic acid	6.41–6.61	Rutin	4000
Quercetin	214–843	Quercetin	36
Kaempferol	22.4–59.7	Kaempferol	4

The study conducted by Qin et al. (2010) analyzed the nutritional composition and flavonoids content in flour obtained from 39 distinct cultivars of tartary and common buckwheat cultivated in China. The result obtained revealed that tartary buckwheat flour had a greater ash content (2.38 %) but lower levels of total starch (70.22 %), amylose (22.32 %), and resistant starch (17.66 %) in contrast to usual buckwheat flour, which had an ash content of 2.17%, total starch of 73.69 %, amylose of 23.01 %, and resistant starch of 18.69 % (p < 0.05). However, the protein, lipid, and crude fiber levels in the tartary buckwheat flour were equivalent to those in normal buckwheat flour. The Mei-Hua-Shan tartary buckwheat flour exhibited the presence of total flavonoids and quercetin in high concentrations, i.e., 22.74 mg/g and 2.38 mg/g, respectively. Janovská et al. (2021) identified catechin, epicatechin, hyperoside, isoorientin, isovitexin, orientin, rutin and vitexin as the primary phenolic compounds present in buckwheat grains cultivated in the Czech Republic from 2019 to 2020. Zhang et al. (2015) conducted a study to investigate the effect of a 72-h germination period on the nutritional contents, chemical composition, anti-nutritional components, and antioxidant activities of buckwheat seeds. The prolonged germination period led to a substantial increase in the concentrations of crude protein, condensed tannins, reducing sugar, total flavonoids, and total phenolics, whereas the levels of crude fat, phytic acid, and trypsin inhibitor activity showed a reduction in their concentrations. During the germination phase, there was a significant increase in the quantities of phenolic compounds like chlorogenic acid, isoorientin, isovitexin, orientin, p-hydroxybenzoic acid, rutin, trans-3-hydroxycinnamic acid, and vitexin, likely due to the activation of phenylalanine ammonialyase. In research conducted by Kreft (2016), it was discovered that buckwheat seeds and flour have antioxidant qualities, prevent premature aging of immune cells, and have beneficial effects on cardiovascular health (Fig. 1).

## 3 Anti-nutritional factors (ANF) and their reduction strategies

Studies have identified that amaranth contains various anti-nutritional compounds like nitrates, oxalates, phytate, protease inhibitors, saponins, and tannins (Aderibigbe et al., 2022). The saponins content in amaranth grains ranges from 0.9 to 4.91 mg/kg, and phytic acid content ranges from 2.9 to 7.9 g/kg (Thakur et al., 2021). In comparison to other cereals like maize and wheat, amaranth contains only trace amounts of protease inhibitors (chymotrypsin and trypsin) with nitrates that are primarily concentrated in the leaves rather than grains, making it a safer choice for consumption (Rastogi and Shukla, 2013). These anti-nutritional substances, like phytic acid serve a protective role in plants as they help to store phosphorous and they inhibit nutrient absorption in humans (Aderibigbe et al., 2022). In humans, phytate is indigestible, and thus, it is not consumed as a source of inositol or phosphate; instead, it binds to proteins, forming complexes that limit nutrient availability. Both oxalate and phytate have been reported to obstruct mineral adsorption and impair starch digestion. The saponins are known to form complexes with minerals like iron and zinc, whereas oxalate binds with calcium, reducing calcium absorption (Jan et al., 2023). The primary ANFs of buckwheat include α-amylase inhibitor, phytic acid, polyphenols, and trypsin inhibitor (Wang and Zhu, 2015).

Various processing methods have been developed to minimize or eliminate the ANFs, thereby increasing the appeal of food grains to consumers. These methods are broadly classified into biological, non-thermal, and thermal methods (Olika et al., 2019). Traditional methods for ANF removal include cooking, dehulling, fermentation, germination, peeling, roasting, soaking, and sterilization (Nasab et al., 2024). Processing by traditional methods have not shown satisfactory results in eliminating or reducing ANFs from agricultural food products (Nasab et al., 2024). In contrast to traditional thermal methods that rely on high temperatures, non-thermal methods minimize heat exposure and protect sensitive nutrients from degradation (Aaliya et al., 2021). Additionally, it has been reported that traditional methods like thermal processing and soaking require substantial amounts of chemicals and extended time to achieve the desired effect (Rehman and Shah, 2005; Nematollahi et al., 2021). For instance, fermentation involves the usage of culture medium, bacteria, and other materials, whereas processes like soaking require a significant amount of water and result in higher labor costs (Tadele, 2015). Although thermal processing is crucial for food production, it has a negative impact on nutritional value due to the formation of unpleasant compounds and diminishes sensory attributes, freshness, and functional properties (Thakur and Kumar, 2017).

Alternatively, modern non-thermal food processing methods serve as a significant advancement in achieving desired improvements like enhancing the palatability of grains, refining their composition, and improving their flavor (Adebo et al., 2021). These methods offer numerous advantages, such as robust processing time, high efficiency at lower temperatures, and adaptability, which have made them a popular choice (Nasab et al., 2024). Additionally, the ability of these methods to selectively target specific anti-nutrients boosts enzyme activity and improves the bioavailability of essential minerals, thus enhancing the nutritional value (Nasab et al., 2024). Non-thermal methods like cold plasma (CP), high hydrostatic pressure (HHP), irradiation (gamma rays), pulse electric field (PEF), pulsed light (PL), and ultrasonication (Nasab et al., 2024). Notably, understanding the biology, chemistry, properties of seed fraction, water solubility, and heat sensitivity of grains, along with the action mechanism of non-thermal methods, allows the selection of appropriate methods for ANFs reduction (Nasab et al., 2024).

## 4 An overview of health aspects using animal and human studies on grain flours proteins and peptides

According to recent studies, proteins derived from whole grains and pulses contain bioactive peptides that exhibit disease-fighting properties (Bouchard et al., 2022). These peptides mechanism of action involves inhibition and alteration of enzymes, vasodilation, reduction of oxidative stress, and regulation of gut microbiota and lipid metabolism (Bouchard et al., 2022).

### 4.1 Amaranth

#### 4.1.1 Animal

Kasozi et al. (2018) conducted a study to examine the effect of calcium levels on s100a1 protein expression, histopathological changes in blood, hepatic and renal tissues, antioxidant activity in male Wistar rats with type 2 diabetic mellitus (T2DM) that were supplemented with amaranth grain (AG). Their findings demonstrated that levels of calcium and s100a1 were diminished in blood, renal, and hepatic tissue during T2DM, but increased after the administration of AG supplements. Moronta et al. (2016) examined how a bioactive peptide obtained from *Amaranthus hypochondriacus* grains influences the immune system in a mouse model of IgE-mediated food allergy. The synthetic SSEDIKE peptide derived from amaranth grain, when administered orally, effectively suppressed the allergic reaction in a mouse model when exposed to milk allergens. This suppression was achieved by inhibiting the secretion of IgE and controlling intestinal inflammation, thereby inhibiting the activation of nuclear factor Kappa light chain enhancer of activated B cells (NF-κB). These effects can be attributed to the induction of local tolerance. In a study conducted by Fritz et al. (2011), it was discovered that when male spontaneously hypertensive rats (SHR) were given *Amaranthus mantegazzianus* seed protein hydrolysates with a hydrolysis degree (DH) of 45 % through intragastric administration, their blood pressure was effectively reduced. Experiments conducted on isolated papillary muscles from hearts and isolated aortic smooth muscle of SHR indicates that the hypotensive effect may be due to a reduction in peripheral resistance. Lado et al. (2015) investigated the effect of supplementing 2.5 % (w/w) *Amaranthus mantegazzianus* protein isolate (AI) into the diet of Wistar rats on their antioxidant status, blood pressure, and lipid profiles. The study revealed that rats on a 1% (w/w) cholesterol (Chol) diet showed a significant increase in the level of triglycerides and cholesterol by 2.3 and 2.5 times, respectively. However, the addition of AI to the CholA group (1 % w/w Chol + 2.5 % w/w AI) showed an 18 % and 47 % reduction in cholesterol and triglycerides levels, respectively.

#### 4.1.2 Human

Calva-Cruz et al. (2023) conducted a pilot trial (non-randomized) to assess the alterations in the structure, function, and composition of the gut microbiota in malnourished children for a duration of three months who were administered with 4 g of popped amaranth. For analysis, serum and samples were taken at both the beginning and end of the trial. The results demonstrated a decrease in the occurrence of *Alistipes putredinis, Bacteroides coprocola*, and *Bacteroides stercoris* bacteria, which are associated with inflammation and colitis. In contrast, there was a rise in the occurrence of *Akkermansia muciniphila* and *Streptococcus thermophiles* bacteria, which are linked to positive health and long life. The results indicated that popped amaranth is a valuable nutritional food that play significant role in combating childhood malnutrition by positively influencing the gut microbiota composition. Chaturvedi et al. (1997) assessed the glycemic index (GI) of grain amaranth, wheat, and rice preparations. Diets consisting of 50 g of carbohydrate were administered, and the levels of blood glucose after meals were measured at various time intervals. The glycemic index was determined for various experimental diets. The diet with the lowest glycemic index was the amaranth-wheat composite flour diet (25:75), with a value of 65.6 %. Subsequently, the wheat diet had a prevalence of 65.7 %, the rice diet had a prevalence of 69.2 %, the amaranth-wheat flour diet with a 50:50 ratio had a prevalence of 75.5 %, and the combination of popped amaranth in milk had a prevalence of 97.3%. Hence, a mixture of 25 % amaranth and 75 % wheat, as well as a combination of wheat and rice, can be classified as low glycemic index (GI) foods. On the other hand, a blend of 50 % grain amaranth and 50 % wheat falls into the category of medium GI food, while a combination of popped amaranth and milk is regarded a high GI diet.

### 4.2 Buckwheat

#### 4.2.1 Animal

Yang et al. (2023) assessed the antidiabetic properties of an antioxidant peptide (Ala-Phe-Tyr-Arg-Trp, AFYRW) derived from tartary buckwheat albumin (TBA) in a mouse model with diabetes produced by a high-fat diet and streptozotocin (HFD/STZ). The research demonstrated that AFYRW inhibited the accumulation of fat in liver cells and reduced levels of triglycerides, while also improving insulin resistance in mice. Wu et al. (2021) examined the effect of soluble dietary fiber (SDF) extracted from tartary buckwheat bran on a high-fat diet and streptozotocin-induced diabetic mice, specifically focusing on reductions in lipid and blood sugar levels. The inclusion of SDF in the meal resulted in reduced levels of fasting blood glucose, improved oral glucose tolerance, increased levels of liver glycogen and insulin, and enhanced lipid profiles in both the serum and liver of diabetic mice. The administration of SDF improved both lipid and glucose metabolism, which led to an increase in the levels of short-chain fatty acids in the cecum, regulated by the phosphorylation of hepatic adenosine-5′-monophosphate-activated protein kinase (AMPK). Li et al. (2019) examined the curative properties of buckwheat husk flavonoids (BHFs) on T2DM db/db mice. Following a nine-week administration by BHFs, there was a considerable reduction in insulin resistance. BHFs have the potential to decrease the concentrations of triacylglycerol (TG), total cholesterol (TC), very-low density lipoprotein cholesterol (vLDL-c), and free fatty acid (FFA) in the bloodstream. They can also reduce TG and TC levels in the liver. Additionally, BHFs can enhance the insulin resistance-related PI3k/AKT2 signal pathway and suppress the levels of sterol regulatory element binding protein-1c (SREBP-1c), fatty acid synthase (FAS), and acetyl-CoA carboxylase-1 (ACC1) mRNA expression in the liver tissue of db/db mice were examined.

Hu et al. (2015) examined the potential of D-Chiro-Inositol (DCI)-enhanced tartary buckwheat extract (DTBE) to address liver damage and hyperglycemia induced by a high fructose (HF) diet in rats. The high-performance liquid chromatography (HPLC) study determined that the purified DTBE contained 34.06 % of DCI. The mice that were given 20 % fructose in drinking water for 8 weeks showed significant increases in abnormal lipid profiles, blood sugar, fatty liver, insulin levels, and oxidative stress, with statistical significance (p < 0.01). In contrast, continuous feeding of DTBE with the high-fat diet led to a reduction in blood glucose, body weight, insulin, low-density lipoprotein cholesterol (LDL-C), total cholesterol (TC), and triglycerides (TG) levels, with these reductions being dose-dependent. Additionally, DTBE also decreased the levels of aspartate aminotransferase (AST), alanine aminotransferase (ALT), lactate dehydrogenase (LDH), and c-reactive protein (CRP) in serum. The treatment also enhanced the hepatic glutathione peroxidase (GSH-Px) and total superoxide dismutase (T-SOD) activity, while lowered the hepatic malonaldehyde (MDA) levels compared to rats on HFD.

## 5 Application of amaranth and buckwheat in food enrichment

**Fig. 2:**
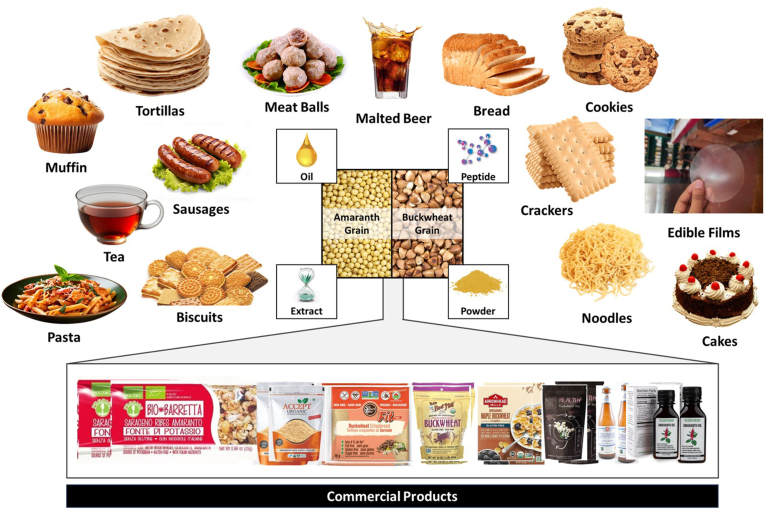
Amaranth and buckwheat fractions and their utilization in functional foods development.

The expanding global gluten-free (GF) market is largely propelled by the increasing prevalence of gluten-related conditions like non-celiac sensitivity, wheat allergy, and celiac disease in developed nations (Foschia et al., 2016). Nevertheless, the rapid increase in sales of gluten-free (GF) products is not only due to the rising number of people with gluten-related disorders, but also because of the growing popularity of these foods among healthy individuals who willingly adopt a GF diet as a lifestyle preference (Prada et al., 2019; Xhakollari et al., 2019). The increasing demand for gluten-free (GF) foods is driving research efforts focused on creating innovative and nutritionally balanced GF food options. Developing novel gluten-free products continues to be a challenge due to the need to enhance their bioactive, technical, and sensory properties. Historically, rice and corn have been the primary components used in gluten-free food products (Martínez-Villaluenga et al., 2020). Nevertheless, in recent times, pseudocereals have surfaced as substitute components in gluten-free formulations due to their inherent gluten-free grain composition. The typical procedure for preparing buckwheat for use in gluten-free foods involves removing the hull, whereas the entire amaranth seed is often milled into flour for use in gluten-free foods (Dziedzic et al., 2016; Guardianelli et al., 2019). Amaranth and buckwheat grains can be fractionated into flour, extracts, peptides, and malt, among other products. This approach can also be utilized for creating functional food items that incorporate various components from different grains; food items include extruded snacks, muffins, cakes, pasta, noodles, cookies, patties, biscuits, bread, malt beer, milk beverages, fermented beverages, sausages, films, and coatings, among others. A significant number of these functional food products are now commercially available (Fig. 2).

### 5.1 Cereal-based foods

Cereal-based foods, such as cakes, bread, crackers, biscuits, cookies, pasta, and muffins are key elements of a well-balanced diet (Fig. 2). They offer significant quantities of carbohydrates and protein. Nevertheless, they do not contain an adequate amount of micronutrients (such as minerals and vitamins), fiber, and phytochemicals. Consumers not only desire meals that provide necessary nutrients but also look for nutrient-dense options to improve their health (Kumar et al., 2023b). Amaranth and buckwheat serve as useful ingredients in cereal-based goods, namely in the form of flour (Table 2). Studies by Gambuś et al. (2010), Schoenlechner et al. (2010), Mariotti et al. (2013), Chauhan et al. (2015), Zieliński et al. (2017), Brites et al. (2019), Bhatt et al. (2021), and Piga et al. (2021) have demonstrated that incorporating amaranth and buckwheat powder into bread, biscuits, cakes, muffins, pasta, cookies, noodles, crackers, rolls, and tortillas can produce gluten-free (GF) foods.

**Table 2:** Application of amaranth and buckwheat grains in cereal-based products and their quality characteristics.

Product developed	Country of study	Type of fraction used	Added amount	Outcomes	Reference
Bread (Gluten-based)	Peru	Amaranth flour (AF)	0−30 %	Compared with wheat bread the highest substitution of AF flour contained bread has increased protein content (12 %) and fiber content (more than 100 %) respectively but decreased carbohydrate content (6 %)	Chaquilla-Quilca et al. (2024)
Bread (Gluten-based)	Romania	AF	5−20 %	The optimization findings showed that the composite flour containing 9.74 % AF of 280 μm particle size mixed in wheat flour (WF) would be the most appropriate combination; The ideal WF-AF bread's physical, textural, and particularly nutritional qualities were improved; Optimal loaves have a mineral value that was up to two times more than that of wheat flour bread	Coțovanu et al. (2023)
Bread (Gluten-based)	Romania	AF	5−20 %	The volume and hardness of bread increased when more than 15 % of the large particle size (PS replaced) wheat flour (WF) was used; The loaf porosity rose when medium and small particle sizes were used as replacements at a rate of 5–15 %; The chroma values exhibited a decline, resulting in darker samples, as the replacement level rose; Substituting whole wheat flour (WF) with different fractions of alternative flours (AF) had an effect on the sensory qualities of bread; This resulted in improved acceptance for bread with larger and medium particle sizes (PS) up to a 10 % substitution rate	Coţovanu and Mironeasa (2022)
Bread (Gluten-based)	Russia	AF	10−40 %	The substitution of 10 % of wheat flour with amaranth flour enhances the quality of wheat bread, as well as augmenting its biological and nutritional content	Kiyashko et al. (2021)
Bread (Gluten-based)	India	AF	5−15 %	The addition of AF increased the moisture content of the bread from 31.06 % to 33.24 %; It also increased the ash content from 0.92 % to 1.51 %, the protein content from 12.17 % to 13.11 %, the fat content from 2.16 % to 2.77 %, and the crude fibre content from 1.11 % to 1.72 %; However, it decreased the nitrogen-free extract content from 52.58 % to 47.65 % and the alkali water retention capacity from 136.00 % to 112.02 %; The analysis of texture revealed that the incorporation of amaranth flour led to an increase in hardness, chewiness, gumminess, springiness, and cohesiveness; The bread samples containing 5 %, 10 %, and 15 % of AF exhibited reduced yellowness (*b* ∗) and increased lightness (*L* ∗) and redness (*a* ∗) values for the crust color; Additionally, these samples had decreased *L* ∗ values and increased a ∗ and b ∗ values for the crumb color; The bread, which substitutes 5 % and 10 % of AF, is both nutritionally and sensorially satisfactory	Nasir et al. (2020)
Bread (Gluten-free)	United States of America	AF	85 %, and 70 %	The addition of amaranth significantly enhanced the solidity of bread made with amaranth-soy 85:15 and 70:30, as well as amaranth-lupin 85:15; The resulting texture was comparable to that of whole wheat bread	Liu et al. (2019)
Bread (Gluten-based)	Spain	AF	10−40 g	Whole amaranth flour can be utilized as a substitute for a portion of wheat flour in bread recipes, enhancing the nutritional content and elevating the levels of dietary fiber, minerals, and protein in the final product; However, there is a noticeable decrease in bread quality when using amaranth flour in amounts ranging from 10 to 20 g per 100 g of flour. Therefore, it is recommended to limit the inclusion of amaranth flour to a maximum of 20 g per 100 g of flour in order to maintain both the quality of the bread and the nutritional benefits provided by this ingredient	Sanz-Penella et al. (2013)
Bread (Gluten-based)	Turkey	Buckwheat flour (BF)	0−30 %	The addition of buckwheat increases the volatile components such as phenylethanol and benzylalcohol; White bread with 20 % BF was considered the most appropriate	Eren and Akkaya (2024)
Bread (Gluten-free)	Spain	BF	50 %	In comparison to the control, BF-fortified dough showed better consistency and elasticity; Increased protein digestibility and reduction in glucose release were observed during *in vitro* starch digestion	Vicente et al. (2024)
Bread (Gluten-based)	Romania	BF	5−20 %	The optimal formulas, in terms of dough rheology and bread qualities, were determined to be 9.13 % BF for big, 10.57 % for medium, and 10.25 % for small PS; The bread manufactured from ideal composite flours with medium and small particle sizes had a significantly greater amino acid content, reaching up to 21.22 %, compared to wheat bread; The bread samples with medium and large particle size (PS) exhibited greater mineral content, with values up to 2.63 times higher than the control sample; The sensory analysis findings indicated that the bread samples with a composition of 9.13 % large and 10.57 % medium particle size were favoured the most by the panellists	Coțovanu et al. (2023)
Bread (Gluten-based)	Poland	BF	10−50 %	The bread made with 20 % buckwheat flour showed a notable rise in protein content; Additionally, the loaves containing buckwheat flour exhibited a significantly reduced whiteness in the crumb, in comparison to wheat bread; An increase in the buckwheat flour content by 10, 20, and 30 % resulted in a significant decrease in crumb hardness, gumminess, and chewiness compared to the other bread samples	Kowalski et al. (2022)
Bread (Gluten-free)	Poland	BF	10−40 %	An increase in the quantity of buckwheat flour used in gluten-free bread recipes resulted in a reduction in the firmness of the bread over time; Incorporating buckwheat flour into gluten-free formulas can have a beneficial impact on the texture of the bread and delay its staling process	Wronkowska et al. (2013)
Bread (Gluten-free)	Poland	BF	10−40 %	The incremental inclusion of buckwheat flour (10−40 %) in bread had a notable impact on the relative augmentation of proteins and microelements, particularly copper and manganese	Krupa-Kozak et al. (2011)
Biscuits (Gluten-based)	India	AF	10−30 %	An increase in antioxidant, flavonoid, and phenolic contents along with good sensory quality was observed with a 15 % AF addition	Poduvachola et al. (2024)
Biscuits (Gluten-free)	India	AF	15−40 g	Based on an assessment of sensory data over acceptance (7.55) showed that 40 g AF was the right amount for making biscuits	Ajay and Pradyuman (2018)
Biscuits (Gluten-based)	Italy	BF	4−12 %	The biscuits made with the greatest proportion of tartary buckwheat whole flour (12 %) had a final rutin concentration of 72 mg per 100 g of dry weight; Consuming 50−60 g of these biscuits daily, which is equivalent to 6–7 pieces, would provide a preventative dose of 40 mg of rutin	Andrea et al. (2010)
Muffins (Gluten-free)	India	AF	10−50 %	The panelists awarded the highest grade to muffins made with a combination of 50% amaranth and 50 % black rice flour, indicating a favorable overall acceptability	Bhatt et al. (2021)
Muffins (Gluten-free)	Poland	BF	25 g	The concentrations of total phenolic compounds, accessible lysine, furosine, free and total fluorescence intermediary compounds (FIC), browning index, and antioxidant capacity in the buckwheat-enhanced gluten-free muffins were higher than those in the control samples.	Zieliński et al. (2017)
Cookies (Gluten-based)	India	BF	50−100 %	As the concentration of BF grew, the spread ratio of cookies dropped; The sensory scores for texture, look, and flavor of cookies declined as the level of BF in the formulation increased; The flavor score decreased dramatically to 5.71 at greater concentrations, perhaps because of the presence of flavonoid component (rutin) in buckwheat flour, which has a bitter taste; The cookies made with the inclusion of 75 % BF and 100 % wheat flour received good scores for overall acceptability, respectively	Chopra et al. (2014)
Cookies (Gluten-based)	India	BF	20−100 %	The hardness and spread ratio of cookies reduced, while non-enzymatic browning (NEB) dramatically increased when the quantity of buckwheat flour in wheat flour rose; The sensory analysis indicated that the cookies were most acceptable when the blending level was set at 40 %	Jan et al. (2015)
Cake (Gluten-based)	Bangladesh	BF	10−40 %	Increasing the proportion of buckwheat flour up to 30 % resulted in the highest quality of buckwheat fortified cakes (BFC) and received a high sensory acceptability score from experienced panelists; From a microbiological perspective, BFC was deemed appropriate for a duration of 09 days	Farzana et al. (2021)
Cupcake (Gluten-free)	Pakistan	BF	10−30 %	The addition of an increasing amount of BF in cupcakes showed the highest sensory score along with a change in proximate composition and physical characteristics; No change in fat or protein contents after 8 days of storage but there was a decrease in moisture content	Abid et al. (2024)
Crackers and Tortilla (Gluten-based)	Egypt	AF	25−100 %	Regarding minerals and amino acids, the analysis showed that the crackers made with formula No. 3 (25 % corn flour/75 % amaranth flour) and the tortilla made with formula No. 2 (50 % corn flour/50 % amaranth flour) contained higher levels of minerals such as iron, calcium, potassium, zinc, magnesium, manganese, copper, and phosphorus; Additionally, these products had higher amounts of essential amino acids like leucine, lysine, and valine; Furthermore, the crackers made with formula No. 3 had the highest content of unsaturated fatty acids and the lowest content of total saturated fatty acids; On the other hand, the tortilla made with formula No. 2 had the highest content of saturated fatty acids and the lowest content of unsaturated fatty acids	Gebreil et al. (2020)

Piga et al. (2021) investigated the impact of incorporating amaranth into gluten-free double-layered flatbreads by partially substituting rice flour (6 %) and starch (6 %) with amaranth. Two distinct fundamental formulations were employed, specifically a combination of rice flour and corn starch, and a combination of rice flour and tapioca starch, with a ratio of 50:50. The study assessed the nutritional attributes, sensory features, texture, and polyphenol composition of the produced flatbreads. The flatbreads enhanced with amaranth exhibited improved color and a notable rise in all polyphenol fractions. However, they had reduced antioxidant activity. After storing the bread for three days, they observed a negative impact on the qualities of starch retrogradation, toughness, and extensibility, particularly when tapioca starch was utilized. Moreover, according to check-all-that-apply (CATA) sensory test, the incorporation of amaranth enhanced the yeast flavour and odour and reduced the softness experienced in the mouth while consuming tapioca-based samples. Whole wheat chapatti is a widely consumed staple meal in India. Banerji et al. (2018) examined the integration of amaranth (*Amaranthus hypochondriacus*) flour (AF) at levels of 20–50 % with wheat flour to enhance rolling characteristics and enhance nutritional value in terms of proteins and micronutrients of Chapatti (Indian flat bread) (gluten-based). After conducting measurements with a textur-o-meter and evaluating the sensory aspects of chapattis, it is recommended to include 40 % AF. The chapatti produced from this mixture exhibited a notable increase in ash content (including iron, calcium, and magnesium), fat, protein, and lysine. Additionally, it demonstrated enhanced protein digestibility when tested *in vitro*. Chandla et al., 2017a, Chandla et al., 2017b examined the characteristics of noodles (gluten-based) made from amaranth starch (AS), heat moisture-treated-amaranth starch (HMT-AS), and corn starch (CS). The noodles were subjected to analysis to determine their amylose content, swelling power, water absorption capacity, color, particle size (PSA), pasting profile (RVA), and thermal (DSC) properties. The HMT-AS noodles had a cooking loss of 20.15 g/100 g, which was lower than the cooking loss of 22.20 g/100 g seen in the AS noodles. The starch noodles made with HMT-AS exhibited a firmer texture, as well as enhanced taste and unique flavour, when compared to AS and CS noodles. Cárdenas-Hernández et al. (2016) found that incorporating amaranth flour into pasta resulted in the highest levels of protein, crude fiber, and ash.

The study conducted by Wronkowska et al. (2019) investigated the impact of adding roasted buckwheat flour or hull (both raw and roasted) on the sensory attributes and consumer satisfaction of commercially produced mixed rye/wheat breads and wheat rolls (both gluten-based). A microbiological safety assessment was performed to evaluate the safety of breads/rolls following storage. The presence of buckwheat ingredients was discovered to have a beneficial impact on the microbiological safety of baked products. Statistically significant variations were seen in 7 out of the total 21 qualities throughout the process of sensory profiling. Nevertheless, the consumer test did not uncover any significant differences between the regular bread and the bread fortified with buckwheat. When the descriptive sensory profiling method was used with the consumer test, significant differentiation was detected only in the wheat rolls that were enhanced with 3% raw buckwheat hull, in comparison to the control rolls. Liu et al. (2022) investigated the impact of common buckwheat bran on the qualities of wheat dough and the quality of noodles (gluten-based). These tests were conducted using several methods such as cooking, texture, rheology, pasting, thermal characteristics, and microstructure analysis. The dough prepared with buckwheat bran exhibited improved qualities compared to the dough made with buckwheat hull. Additionally, the noodles made with buckwheat bran demonstrated higher quality than those made with buckwheat hulls. The inclusion of common buckwheat bran or hull led to a reduction in the starch pasting qualities as a result of water absorption by the fiber, which impeded the swelling of starch granules. Incorporating either 4 % buckwheat bran or hull into the dough led to the development of desirable attributes such as firmness and resilience. The tensile properties of buckwheat bran noodles were determined to be better in contrast to buckwheat hull samples, even though the ΔH and cooking loss of buckwheat bran noodles were lower. The study conducted by Szawara-Nowak et al. (2016) evaluated the effect of a laboratory process that imitates the chemical and physical changes that happen during digestion in the stomach and small intestine on the antioxidant capacity and bio-accessibility of phenolic compounds in 16 varieties of buckwheat boosted wheat bread. After digestion, the bio-accessible phenolics constituted approximately 90 % of the total phenolics and showed a strong correlation with the global antioxidant response (GAR) values of buckwheat-enriched wheat loaves. The results demonstrated that the *in vitro* digestion process is the critical stage responsible for the release of a substantial quantity of phenolic antioxidants.

### 5.2 Meat-based foods

Suychinov et al. (2023) analyzed how the addition of amaranth flour to meat patties affected their chemical composition. For assessment, four distinct types of meat patties were prepared, with amaranth flour at concentrations of 5 %, 10 %, and 15 % as a substitute for beef, and compared with a control sample containing no additives. The finding suggests that the substitution of beef with 10 % amaranth flour could enhance the carbohydrate and lipid content and improve the water-holding capacity and mineral composition of the patties. Pérez-Ramírez et al. (2023) assessed the nutritional, nutraceutical, and antioxidant capabilities, along with the physicochemical characteristics, of minced tilapia fillet gels enriched with varying amounts (0 %, 2 %, 4 %, 8 %, and 10 % w/w) of amaranth seed or sprout flours. The inclusion of amaranth seed resulted in a substantial increase in dietary fiber content, ranging from 1.25 to 1.75 times higher. Similarly, the addition of sprout flours led to a large rise in dietary fiber content, ranging from 1.99 to 3.21 times higher. Tilapia gels supplemented with 10 % amaranth seed flour exhibited a significant presence of extractable dihydroxybenzoic acid and cinnamic acid. Conversely, the inclusion of 10 % amaranth sprout flour resulted in a rich and diverse array of bioactive components, primarily amaranthine and bound ferulic acid. The suitability of amaranth flour as a substitute for corn starch in sausage production was assessed by Muchekeza et al. (2021). The flour exhibited a notable disparity (p < 0.05) in terms of protein, carbs, moisture, ash, and fat content. There were notable differences in functional qualities, with the exception of emulsion stability and pH. The thermal characteristics of flours exhibited a statistically significant difference (p < 0.05). The study conducted by Ostoja et al. (2002) involved the addition of a mixture of unrefined and puffed amaranth seeds to a batter produced from animal fat. The purpose was to investigate the impact of this addition on the quality of the batter. The meat-fat batter was produced using Class II pork. The findings indicated that utilizing grit derived from unprocessed amaranth seeds had a beneficial impact on the stuffing's ability to retain water, thereby leading to a reduction in cooking losses when canned. Enhanced water retention capacity led to an enhancement in the softness, juiciness, and flavour of preserved meat.

Salejda et al. (2022) conducted a study to assess the impact of adding buckwheat husk (BH) to frankfurter-type sausages with the dual aim of improving sausages nutritional value and minimizing buckwheat waste. On comparing sausages without BH (butylated hydroxytoluene) to sausages with 3 % BH, it was found that the sausages with BH had larger cooking losses but lower storage losses. After a two-week storage period, BH enhanced the firmness of the sausages. The increasing incorporation of BH led to a reduction in the L* and b* values. The alteration in color led to a decrease in consumer acceptance. The addition of BH did not result in a decrease in protein digestibility. The study revealed the direct relationship between the amount of BH added and the total amino acid content, which ranged from 161.8 mg/kg to 228.0 mg/kg. Additionally, BH enhanced the level of calcium, magnesium, manganese, and potassium. Atambayeva et al. (2023) assessed the impact of germinated green buckwheat and its flour (GGBF) on the shelf-life, quality, and safety of mixed chicken thigh and horsemeat patties. The addition of GGBF showed significant improvements (p ≤ 0.05) in fat and protein levels, moisture content, cooking efficiency and fat retention, and overall phenolic content. Table 3 presents the utilization of different portions of grains in the creation of food products mostly made from meat.

**Table 3:** Application of amaranth and buckwheat grains in meat-based products and their quality characteristics.

Product developed	Country of study	Type of fraction used	Added amount	Outcomes	Reference
Turkeys' pâtés	Poland	Amaranth seed	6,8, and 10 %	The results suggest that substituting wheat rolls with seeds significantly improved the nutritional composition (protein, ash, fat, polyunsaturated and polyene acid content) of pâtés in all groups; Additionally, this substitution led to a decrease in the proportion of saturated fatty acids and the ratio of omega-6 to omega-3 acids (3:1), resulting in pâtés with increased firmness and brightness; Furthermore, the use of seeds also improved the microbiological safety of the pâtés; The pâté with 8 % plant additions received the highest ratings for taste appeal and bonding	Augustyńska-Prejsnar et al. (2022)
Goat meat nuggets (gluten-free)	India	Amaranth flour	1.5, and 3 %	The inclusion of amaranth flour (1.5 % and 3 %) had a significant impact (p < 0.05) on the stability of the meat batter emulsion; Incorporating amaranth into meat products resulted in a significant (p < 0.05) increase in dietary fibre content; The addition of pseudo cereal has an impact on the rheological properties of the beef batter	Verma et al. (2019)
Chicken burger	Italy	Amaranth seed	1, and 2 %	Enhanced lipid stability over the storage period; The sensory quality features of chicken burgers were not statistically significant; Even in certain instances (such as burgers containing 2 % amaranth), the overall acceptability was rated higher than that of the control burgers	Longato et al. (2017a)
Chicken nuggets	Iran	Amaranth flour	0,50, and 100 %	The addition of amaranth flour substantially enhanced the mineral, fiber, fat, and protein content of the nuggets; All nuggets containing amaranth flour exhibited a higher pH level compared to the control group; The emulsion stability of the paste was greatest in samples containing amaranth flour in all layers; The texture profile analysis (TPA) characteristics and cutting force shown a direct correlation with the increasing quantity of amaranth in each layer.; Nevertheless, the use of amaranth flour resulted in the darkening of the nuggets; The sensory evaluation of nuggets revealed that replacing wheat flour with amaranth flour in nuggets did not result in a significant impact on the overall acceptability of the product	Tamsen et al. (2018)
Semi-soaked sausage	Kazakhstan	Buckwheat flour	4−12 %	The addition of hydrated buckwheat flour, up to 6 % of the raw meat mass, positively impacts the physical and chemical, functional and technological, structural and mechanical, and organoleptic properties	Yessengaziyeva et al. (2023)
Fried pork meatballs	Poland	Buckwheat hull extract	0.05−0.5 %	Buckwheat hull extract exhibited the greatest capacity to regulate peroxide and 2-thiobarbituric acid reactive substances (TBARS) values; Additionally, it displayed superior 2,2-diphenyl-1-picrylhydrazyl (DPPH) free radical scavenging activity and Fe(II) ion chelating ability compared to butylated hydroxytoluene (BHT)	Hęś et al. (2017)
Fish fillets	China	Buckwheat peptides (BP)	0.5−2 %	The groups treated with TBPs exhibited lower rates of quality degradation in terms of physicochemical, bacteriological, and sensory parameters, as compared to the control group; The shelf life of control tilapia fillets was determined to be 4 days based on the analysis of total volatile base nitrogen content, total viable counts, and sensory scores; In contrast, the shelf life of TBP-treated fillets was found to be 8 days, which is twice as long as that of the control group	Zhang et al. (2019)
Fish fillets	China	Buckwheat extract	0.5−1.5 %	The addition of 1.5 % extract increased the shelf life to 15 days.	Yang et al. (2019)
Emulsion-type sausage	Korea	Buckwheat powder	0−3 %	Higher quantities of additional buckwheat powder resulted in elevated levels of moisture (p < 0.05), ash content (p < 0.05 or >0.05), and cooking output (p < 0.05); The sausage samples exhibited reduced protein and fat contents (p < 0.05) as the levels of added buckwheat powder increased. The sensory characteristics, with the exception of softness, showed significantly higher scores (p < 0.05) in sausages containing 3 % buckwheat powder, as determined by a panel of evaluators	Lee et al. (2018)

### 5.3 Beverage's enrichment

In the present day, consumers are no longer satisfied with flavour alone and are seeking beverages that are both high in quality and rich in nutrients. As a result, non-alcoholic beverage firms have been focusing on producing low- or no-calorie drinks and placing importance on using organic components in their products (Dini, 2019). Functional beverages are a significant category of functional food products as they allow for the inclusion of beneficial nutrients and bioactive substances. These beverages serve to maintain human hydration and offer various effects such as anti-aging, energy enhancement, relaxation, and attractiveness enhancement (Dini, 2019).

The researchers Milán-Carrillo et al. (2012) utilized amaranth grains that had been processed through extrusion to create a beverage with immediate functional properties. Higher acceptance as accorded for samples containing amaranth in comparison to those without amaranth. Kunu, a traditional beverage (non-alcoholic) from Nigeria, was prepared by fermenting amaranth grain for varying durations. It was then compared to the widely recognized Kunu beverage made from sorghum, as described by Isaac-Bamgboye et al. (2019). Results from the sensory assessment revealed that amaranth-containing kunu had a higher level of acceptance than sorghum-containing kunu after a 48-h fermentation period. In addition, Amaranth-Kunu showed an increased concentration of ash, fat, and protein as compared to Sorghum-Kunu. The study conducted by Espino-González et al. (2018) evaluated key athletic performance characteristics in cyclists using a randomized crossover design. The participants were administered either an amaranth-derived beverage or a commercially available beverage. All tested variables showed no significant differences, apart from time-trial preference, where the amaranth beverage demonstrated higher performance than the commercial beverage. The amaranth beverage was prepared by mixing amaranth grains (88.8 g), sugar (41.3 g), and sodium chloride (0.35 g) in 1 L of water. Subsequently, it was infused with grape or orange flavor. The objective of this was to attain a protein concentration of 1.5 %, a carbohydrate content of 10 %, and an electrolyte concentration of 0.35 g/L (specifically sodium chloride). The higher caloric content of amaranth beverages (52.48 kcal per 100 mL) compared to commercial beverages (24 kcal per 100 mL) is considered to be the reason for the differences observed in time-trial performance.

Mousavi et al. (2023) enhanced the formulation of gluten-free beverages made from buckwheat and lentil, which were fermented using *Lactobacillus plantarum* and *Bifidobacterium bifidum.* The results indicated that the optimized probiotic beverage had a lactic acid acidity of 0.25 %, a pH of 5.7, a total solids content of 7.9 %, an ash content of 0.4 %, a 2,2-diphenyl-1-picrylhydrazyl (DPPH) value of 41.02 %, a phenol component concentration of 26.96 mg gallic acid equivalents (GAE)/mL, and a probiotic count of 8.65 log CFU/mL. The optimized beverage exhibited specific sensory characteristics on the 15th day of being stored in a refrigerator. Zielinska et al. (2013) examined the use of buckwheat hulls for making buckwheat husk tea. The buckwheat hull is composed of rutin, quercetin, and C-glucoflavones. Nevertheless, the overall phenolic concentration was significantly reduced in comparison to that found in green tea leaves. Rutin and vitexin were the main flavonoids detected in buckwheat husk tea. Green tea exhibited a higher antioxidant capacity and inhibitory effect against the formation of fluorescence-advanced glycation end products compared to buckwheat husk tea. Beer, a worldwide popular fermented beverage, has driven the research into using amaranth and buckwheat for producing gluten-free high-quality beverages similar to beer (Martínez-Villaluenga et al., 2020). Table 4 exhibited the malted beer that was created using amaranth and buckwheat grains.

**Table 4:** Application of amaranth and buckwheat grains as malt beer and their quality characteristics.

Raw materials	Country of study	Finished product characteristics	Reference
60% of barley malt with 40% of amaranth malt	Italy	Reduced extraction efficiency results in lower yields of the extract, as well as smaller volumes of the final beers with a pH of 4.0	Buiatti et al. (2018)
100% amaranth malt	India	The alcohol percentage of the beer rises as the fermentation period lengthens and the amount of reducing sugars decreases; Additionally, an increase in fermentation days leads to a drop in the beer's pH and tannin content	Malganve et al. (2022)
70% of barley malt with 30% dehulled amaranth seeds, flakes and popping	Spain, and Poland	The use of amaranth significantly elevated the Mg^2+^ to Ca^2+^ ratio, as well as the levels of Zn^2+^ and Mg^2+^ in the wort	Bogdan et al. (2020); Cadenas et al. (2021)
100% buckwheat malt	Slovenia	The fermentable carbohydrate level in the buckwheat wort was similar to that of barley; The amino acid composition of the buckwheat wort closely resembled that of barley; The sensory evaluation of the buckwheat beverage indicated a higher level of perception and demonstrated widespread approval	Deželak et al. (2014)
100% buckwheat malt	Ireland	The yield of the buckwheat wort extract was reduced, resulting in a final extract yield of 54.5 %; The gas chromatography (GC) study of the beer showed typical levels of esters, which contribute to the fruity taste of the beer; A relatively little amount of fusel alcohols, when compared to a conventional wheat beer, was identified; The analysis revealed a significant presence of ethyl caprinate, which imparts a coconut flavor, as well as lauric acid, which contributes to a fatty odour; The sensory research revealed that the buckwheat beer was deemed satisfactory in terms of its aroma, taste purity, texture, tingling sensation, and bitterness	Phiarais et al. (2010)
20 and 40% buckwheat malt	China	The results unequivocally demonstrate that the primary characteristics of beer quality were mostly unaltered when substituting 20 % or 40 % of malted barley with tartary buckwheat malt; The sensory study results indicated that the buckwheat beers were deemed satisfactory, particularly in terms of their odour, mouthfeel, and taste; The rutin and total flavonoid content in the buckwheat beers were significantly influenced by the mashing routines	Deng et al. (2019)
55% (w/w) barley malt and 45% (w/w) buckwheat malt	Brazil	The utilization of buckwheat malt resulted in a significant increase of over 89 % in the protein content, a phenomenon not observed with the use of other additives; Furthermore, it exhibited superior colloidal stability during the storage duration, which is linked to a remarkable four-fold decrease in gluten content	Brasil et al. (2021)

### 5.4 Films/coatings

The growing usage of bioactive composite films and coating in the food packaging industry is due to their antioxidant and antibacterial properties, which contribute to food safety and improved quality by curbing the growth of harmful microorganisms and delaying of oxidation process that could lead to spoilage (Kumar et al., 2023b). These packaging materials are generally categorized into categories like antioxidant films/coatings, antibacterial films/coatings, or other active packaging materials based on their specific bioactive compound, as all are generated from bio-based materials incorporated with bioactive components like antimicrobial peptides, essential oils, and plant extracts. The incorporation of bioactive components from polysaccharides, starch, and protein hydrolysate of amaranth and buckwheat has enhanced the functionality of smart functional films and ingestible coatings (Table 5). The films mentioned in these studies utilize bioactive chemicals as a natural addition, exhibiting various mechanical, physical, barrier, structural, and functional properties (Condés et al., 2015; Chandla et al., 2017a, Chandla et al., 2017b; Sindhu and Khatkar, 2018; Tian et al., 2021; Yadav et al., 2023).

**Table 5:** Applications of amaranth and buckwheat grain edible films/coatings in food products.

Product developed	Country of study	Type of fraction used	Added amount	Outcomes	Reference
Starch-protein hydrolysate	Czech Republic	Amaranth flour protein hydrolysate	10, and 30 %	After being dried for 5 min at temperatures of 23, 30, or 40 °C, a protective film formed on the strawberries; The strawberries were held at temperatures of 7 ± 1.5 °C or 23 ± 1.5 °C; The protective coating, which included 30 % glycerol, resulted in the least amount of mass loss due to humidity evaporation at both storage temperatures	Mokrejš et al. (2009)
Steric acid/glycerol	Brazil	Amaranth flour	100 g	The improved coating demonstrated superior efficacy in preserving the firmness of strawberries; Additionally, the optimized coating led to a slight elevation in the chromaticity ratio (a/b) over the course of storage	Colla et al. (2006)
Chitosan-based	China	Buckwheat starch	3 g	When compared to the control samples, the mutton that was wrapped with the active film showed a decrease in pH, as well as reduced levels of total volatile basic nitrogen and thiobarbituric acid reactive substances during the entire storage duration; Additionally, the storage time of the mutton was extended by 2 days at a temperature of 4 ± 1 °C	Tian et al. (2021)
Starch-based	Korea	Buckwheat starch	15, and 30 g	A film was used to coat the packing of freshly cut mushrooms, and this film showed antimicrobial properties against *Listeria monocytogenes;* As a result, there was a decrease of 0.86 log CFU/g in the bacteria count after 6 days of storage	Kim and Song (2018)
Polysaccharides-based	China	Buckwheat polysaccharides	5, 10, and 15 g/kg	The shelf life of tilapia (*Oreochromis niloticus*) fillets was prolonged by 8–10 days	Wang et al. (2018)

Yadav et al. (2023) conducted a study focusing on the modification of Amaranthus starch via oxidation and heat-moisture treatment (HMT) to develop edible films. The developed films were then assessed for their mechanical and physicochemical properties. The result obtained from the study revealed that the carboxyl content of the native starch and HMT starch was similar, but oxidized starch had a higher greater carboxyl content (0.1 COOH/100GU). The samples of modified starch showed increased solubility, i.e., 76.44 %, in comparison to native starch solubility, which was 62.58 %, while the swelling capacity was higher in native starch at 50.43 g/g. Further, the gelatinization temperature varied for native starch, oxidized starch, and heat moisture-treated starch, which was measured to be 75.09 °C, 76.10 °C, and 89.36 °C, respectively. Edible films were produced from these starches and evaluated for their mechanical and physicochemical characteristics. Both modification techniques enhanced the tensile strength of amaranth starch films. However, only the addition of HMT resulted in an increase in the permeability of water vapor, whereas oxidation had the opposite effect. Coelho et al. (2022) conducted a study on the creation of films made from alginate that included phenolic chemicals isolated from *Amaranthus cruentus* grain. Various solvents were used in the process. The films were produced using alginate, glycerol, and amaranth grain phenolic chemicals at different quantities. A central composite rotatable design (CCRD) experiment was executed to assess the impact of different factors of the films, such as hydrophobicity, mechanical properties, moisture content, optical properties, solubility, thermal properties, and water vapor permeability. The finding of the study revealed that ethanol was an effective solvent for the extraction of antioxidants and phenolic compounds from amaranth grain. The production of alginate films with amaranth phenolic compounds was effectively achieved. This research provides valuable insights for customizing the composition of alginate films with amaranth phenolic compounds based on their intended usage in culinary applications. Elizondo et al. (2009) conducted a study focused on developing biodegradable films formulated using *Amaranthus cruentus* flour combined with poly (vinyl alcohol) (PVA), specifically PVA 325. In this study, films were created with PVA 325 content varying from 10 % to 50 %. The finding revealed that increasing the PVA 325 content improved all the mechanical properties and reduced water solubility, i.e., 44% for films with 50 % PVA.

The pH-sensitive films were fabricated by the casting technique, utilizing buckwheat starch (BS) as the primary material at a concentration of 5 % (dry basis) (Thakur et al., 20203). Citric acid (CA) was employed as a crosslinker, with a weight percentage ranging from 1 % to 5 % relative to the dry starch. Additionally, natural rose petals extract (RE) was incorporated as a pH indicator, constituting 5 %–15 % of the total solution volume. The study determined that film was deemed preferable and might serve as intelligent packaging for monitoring the quality and safety of food (Thakur et al., 2023). Sindhu and Khatkar (2018) discovered that edible films produced from heat-moisture-treated buckwheat starch exhibited increased tensile strength and improved moisture barrier properties.

## 6 Pre-clinical and clinical studies of amaranth and buckwheat food products

### 6.1 Animal studies

Olugbuyi et al. (2023) conducted a study on the glycemic index/load of bread made from flours derived from wheat, mature green plantain, and amaranth grain on Wistar rats. Rats who were fed a diet consisting of 60 % wheat, 20 % plantain, and 20 % amaranth bread (WPAB) had the lowest increase in glucose levels after eating, compared to rats that were fed glucose (used as the reference control) which had the highest increase in glucose levels. The glycemic index and glycemic load exhibited the lowest values (37.56 and 14.15 %) in rats that were fed with WPAB, while they reached their highest levels in rats that were given glucose (100 and 50 %), respectively. Omoba et al. (2022) assessed the effect of extruded snacks enriched with amaranth and fortified with shallot on hematological indices, blood glucose levels, and the activity of carbohydrate-degrading enzyme in streptozotocin induced diabetic rats. The study findings demonstrated that rats who consumed snacks exhibited beneficial effects on their blood-related parameters, reduced the increase in renal and hepatic enzyme activity, and showed a decrease in α-glucosidase/α-amylase activities.

Wang et al. (2023) produced fermented buckwheat milk (FBM) using probiotic lactic acid bacteria. An investigation was carried out to assess the influence of FBM on body weight, gut microbiota, and short-chain fatty acids (SCFA) in mice. The use of FBM showed a substantial impact in effectively managing weight gain. Furthermore, the mice that were given FBM had an enhancement in the variety of microorganisms present in their intestines. Conversely, the levels of six short-chain fatty acids (SCFA) in the FBM group were notably elevated compared to the other two groups (Table 6). These findings will offer valuable insights into the probiotic functions and the development of buckwheat-based products as substitutes for dairy. Wu et al. (2022) employed tartary buckwheat straight noodles (TSNw) made from the entire plant to manage type 2 diabetes mellitus (T2DM) in Wistar mice. Folllwing a six-week dietary intervention, serum metabolic analysis of T2DM rates demonstrated that TSNw significantly decreased the liver enzymes (glutamic-pyruvic transaminase and glutamic oxaloacetic transaminase), triacylglycerols, creatinine, uric acid, total cholesterol, low-density lipoprotein cholesterol, and urea. Additionally, it increased the levels of while increasing total protein, and high-density lipoprotein cholesterol and improved the activity of glutathione peroxidase and superoxide dismutase. Suzuki et al. (2015) investigated the potential toxic effects of dough containing high concentrations of rutin from the tartary buckwheat variety 'Manten-Kirari.' In this study, rats were administered with doses of 10,000 mg/kg and 5000 mg/kg of wheat, respectively, for acute and subacute toxicity studies. The results revealed no significant toxic effect at the dosage of 5000 mg/kg.

**Table 6:** Animals studies to check the beneficial health effects of amaranth and buckwheat grains-derived products.

Country of study	Animal model/health condition	Types of food	Added amount	Health benefits	Reference
Venezuela	Sprague Dawley Rats/Induced hyperglycaemia and hyperlipidaemia	Wholemeal bread with amaranth	Amaranth flour 100, and 200 g/kg	The rats who consumed diets with 100 g/kg and 200 g/kg had the lowest glucose levels; However, there were no significant differences (p > 0.05) between the groups of rats with high blood lipids and glucose (E); The concentrations of triglycerides decreased in the 100 g/kg and 200 g/kg treatments compared to the control diet (CD) in rats with increased blood lipids and glucose (E); Animals at a concentration of 100 g/kg exhibited a decreased total cholesterol level compared to animals with normal concentrations; The levels of high-density lipoprotein cholesterol significantly increased in the groups receiving 100 g/kg and 200 g/kg (p < 0.05); Conversely, the levels of very low-density lipoprotein cholesterol and cardiac risk index decreased (p < 0.05)	Sánchez-Urdaneta et al. (2020)
Japan	Sprague-Dawley rats/Induced hypercholesterolemia	Buckwheat protein product (BP)	Buckwheat protein 20 %	Feeding rats cholesterol and consuming BP at a net protein level of 20 % for 13 days resulted in significant decreases of 32 % and 25 % in blood cholesterol levels, compared to consuming casein (p < 0.05)	Tomotake et al. (2007)
China	C57BL/6 rats/Normal	Buckwheat fermented milk	Not specified	Administration of buckwheat fermented milk effectively prevented the elevation of lipopolysaccharide levels in the colon and the increase in antioxidant indices in rats fed a high-fat diet (HFD); The consumption of buckwheat fermented milk can greatly increase the presence of *Firmicute*s bacteria and reduce the presence of *Bacteroidetes* bacteria, in comparison to a high-fat diet supplemented with fermented milk (HFDFM); Additionally, the concentration of short-chain fatty acids (SCFAs) in the high-fat diet supplemented with buckwheat fermented milk (HFDBFM) was higher than in the other groups	Zhou et al. (2019)
United States of America	Crossbred piglets/normal	Buckwheat flour	Not specified	*Lactobacillus delbrueckii, Lactobacillus crispatus*, and *Fusobacterium* sp. were more abundant in the small intestine of piglets fed a plant-based formula compared to those fed a dairy-based formula. *Bacteroides nordii*, *Enterococcus* sp., *Lactobacillus crispatus*, *Prevotella* sp., *Ruminococcus lactaris*, *Bacteroides nordii*, *Eisenbergiella* sp., *Lactobacillus crispatus*, *Prevotella* sp., and *Akkermansia muciniphila* were more abundant in the large intestine of piglets fed a plant-based diet compared to those fed a dairy-based diet; Circulatory cytokines, magnesium, triiodothyronine (T3), thyroxine (T4), thyroid stimulating hormone (TSH), vitamin D, vitamin K, and IgE levels were similar in all piglets, regardless of their dietary group	Gurung et al. (2023)
Italy	Spontaneously Hypertensive rats/Spontaneously hypertensive	Buckwheat sprouts pasta (BSP)	30 % buckwheat sprouts	The findings indicated that SHR given BSP demonstrated elevated plasma concentrations of the endogenous vasodilators bradykinin (BK) and nitric oxide (NO), reduced levels of the vasoconstrictor endothelin-1 (ET-1), and enhanced antioxidant capacity	Merendino et al. (2014)

### 6.2 Human studies

The glycemic indices of optimal multigrain snack bar products made from amaranth were assessed on a group of healthy, non-smoking, non-diabetic individuals (both male and female) with body weights ranging from 28 to 47 kg and a body mass index between 22 and 24.8 kg per square meter (Olagunju et al., 2022). The multigrain snack product (MS) was developed by blending pre-fermented flours of amaranth, acha, and pearl millet in different proportions. This was done after optimizing the ratios using response surface methodology. Two specific ratios were used, namely SB1 (90: 5: 5) and SB2 (47.98: 26.68: 25.34). For comparison, a control sample from the oat snack bar market (SBC) was employed. The results revealed that the newly developed snacks, especially SB1, showed a medium glycemic load (GL) and low glycemic index (GI) values. The low postprandial glucose readings of the subjects were as follows: 145, 130, and 109 mg/dL at 0 min, 163, 147, and 154 mg/dL at 15 min, and 144, 129, and 141 mg/dL at 120 min for SB1, SB2, and SBC correspondingly. These findings suggest that snacks are unlikely to raise blood glucose levels and can be considered a healthy option for both diabetic and non-diabetic individuals. The study by Jamka et al. (2021) assessed the impact of rapeseed and amaranth oils on atherosclerosis-related parameters in obese or overweight individuals. In this randomized cross-over trial, 44 participants were instructed to consume 20 mL of both oils. These consumption periods lasted for three weeks each and were conducted consecutively. A washout period, which lasted the same amount of time as the intervention, was introduced between the two consumption periods. The study measured various parameters including adiponectin, apolipoproteins (Apo) A1, B, and E, oxidized low-density lipoprotein, tumor necrosis factor-alpha, as well as markers of insulin and glucose regulation. Amaranth oil showed a modest rise in adiponectin levels relative to rapeseed oil, but it adversely affected ApoB levels and the ApoB/A1 ratio. Dus-Zuchowska et al. (2019) conducted a comparison between this study, examining the impact of supplementing with amaranth oil (AmO) and rapeseed oil (RaO) on the development of early markers of atherosclerosis (AT) and lipid profile in individuals who were obese or overweight. A randomized, double-blinded cross-over experiment was conducted, wherein subjects ingested 20 mL of AmO in the initial phase and 20 mL of RaO in the subsequent phase, with a transition occurring after the washout time. Before and after dietary interventions, the serum levels of adhesion molecules (sP-selectin, sVCAM-1), high-sensitivity C-reactive protein (hsCRP), asymmetric dimethylarginine (ADMA), and lipid profile were assessed. Furthermore, anthropometric parameters were assessed. Compared to the RaO group, the AmO group showed a marked increase in low-density lipoprotein (LDL) cholesterol and total cholesterol. These evidences suggest the AmO consumption could increase the risk of cardiovascular issues in individuals who are obese or overweight.

Nishimura et al. (2016) assessed the impact of tartary buckwheat noodles, which are rich in rutin, on decreasing arteriosclerosis, providing antioxidant benefits, and promoting weight loss. 144 adult individuals with an atherosclerosis index (AI) of 2.25 ± 0.65 were randomly assigned to there were two groups in the study: one group drank goods with rutin-rich tartary buckwheat, while the other group consumed a placebo. No notable disparities were observed across the groups in relation to the atherosclerosis index and levels of oxidized-low density lipoprotein (ox-LDL). During the eighth week, the active test meal group showed a significant reduction in level of body mass index (BMI), body weight (BW), and thiobarbituric acid reactive substance (TBARS) compared to the placebo group (p = 0.027, p = 0.030, respectively). Moreover, by the 4-week, the active test meal group showed a notably lower body fat percentage (BFP) relative to the placebo group (p = 0.038). Therefore, consuming tartary buckwheat, which is high in rutin, may be beneficial for managing body weight as a result of its antioxidant characteristics. To investigate the potential positive effects of dietary changes on specific indicators of atherosclerosis, researchers conducted a one-month dietary intervention study on normal-weight patients who were already taking statin therapy (Stokić et al., 2015). The study sought to assess the efficacy of buckwheat-enriched wheat bread in reducing hyperlipidemia (Stokić et al., 2015). Results showed that a notable reduction in total cholesterol and LDL-cholesterol, as well as the LDL/HDL cholesterol ratio, was seen (Table 7).

**Table 7:** Human studies to check the beneficial health effects of amaranth and buckwheat grains-derived products.

Country of study	Subject condition	Types of food	Added amount	Health benefits	Reference
Ethiopia	Anemic children (2-5 years-old)	Amaranth flour bread	70 %	The prevalence of anemia was substantially lower in the amaranth group (32 %) compared to the maize group (56 %); The beta coefficient for estimating hemoglobin concentration was substantially greater in the amaranth group compared to the maize group; The incidence of iron deficiency anemia is considerably reduced in the amaranth group	Orsango et al. (2020)
Brazil	Moderate hypercholesterolemic men (30–65 years)	Amaranth snacks	50 g	Amaranth and placebo did not show any disparities in terms of total cholesterol (TC), low-density cholesterol (LDL-c), and triglycerides (TG); However, amaranth snacks notably decreased high-density cholesterol (HDL-c)	Chávez-Jáuregui et al. (2010)
Mexico	Type 2 diabetes mellitus (T2DM)	Amaranth flour bread	20 %	Serum markers associated with obesity, including leptin, resistin, and visfatin, exhibited a drop in all groups; Additionally, the cardiovascular risk biomarker plasminogen activator inhibitor 1 (PAI-1) also fell following the three-month therapy period.	Gómez-Cardona et al. (2017)
China	Type 2 diabetes mellitus (T2DM) women (5–20 years)	Buckwheat noodles	Not specified	The consumption of buckwheat-based food in the TB intervention group resulted in a significant decrease in the relative changes in urinary albumin to creatinine ratio (UACR) and urea nitrogen (UN) compared to the diet control group after 4 weeks (p < 0.05); There was no noticeable effect on blood glucose levels during the 4-week study. These findings provide support for the hypothesis that replacing staple food with buckwheat may help alleviate renal dysfunction in patients with type 2 diabetes mellitus (T2DM)	Qiu et al. (2016)
Sweden	Healthy female day-care centre staffs (mean age 46 years)	Buckwheat cookies	970 g	Significant decreases in both total serum cholesterol (p < 0.001) and HDL-c (p < 0.001) were observed throughout the trial period, accompanied by an improvement in lung vital capacity (p < 0.001).	Wieslander et al. (2011)
Italy	High risk cardiovascular men and women (mean age 51 years)	Buckwheat bread, pasta, biscuits, crackers	Not specified	The consumption of buckwheat products led to a notable improvement in various health markers, including a decrease in total cholesterol by 4.7 %, low-density lipoprotein cholesterol by 8.5 %, triglycerides by 15 %, glucose by 5.8 %, and insulin by 17 %. These improvements were observed regardless of age, sex, body mass index, and hypertension	Dinu et al. (2017)

## 7 Amaranth and buckwheat as animal feed enrichment

To adequately nourish the global population by 2050, there is a need for a substantial rise of 60–70% in the intake of meat and milk. The majority of the expansion will be ascribed to emerging economies (Kumar et al., 2023b; Wadhwa et al., 2015). The future will witness a surge in the demand for cattle products, driven by the rise in income, population, and urbanization. Livestock exhibits a remarkable growth rate in developing nations, making it one of the prominent subsectors in agriculture. This will lead to a substantial surge in the demand for animal feed. The scarcity of premium feed greatly impedes the cattle production in underdeveloped nations. Functional feed holds significant promise in the aquaculture sector (Kumar et al., 2023b). Hence, it is imperative to decrease or eradicate animal ingredients from functional feed formulations and instead replace them with more cost-effective plant products. However, the type of plant proteins, lipids, carbohydrates, and their exact composition in the formulation has been associated with economic, environmental and health concerns (Kumar et al., 2023b). Amaranth and buckwheat grains have a significant impact on the development of functional chicken feed, as outlined in Table 8.

**Table 8:** Application of amaranth and buckwheat grains in chicken feed and their health characteristics.

Chicken condition	Country of study	Type of fraction used	Added amount	Health benefits	Reference
7-day old broiler	Australia	Amaranth grain	200, 400, and 600 g/kg	Increasing amounts of raw amaranth in the meal resulted in a significant decrease (p < 0.01) in weight increases and feed intake; The feed and weight gain values were comparable in birds' fed diets with 0 and 200 g/kg of amaranth; However, they significantly increased (p < 0.01) beyond the 200 g/kg level of inclusion; It is possible to incorporate grain levels up to 400 g/kg in broiler diets without any negative impact on performance	Ravindran et al. (1996)
67-weeks old Hy-line W-36 white leghorn laying hens	Iran	Amaranth grain	5,10, and 15 %	The ingestion of amaranth resulted in a reduction in blood glucose, cholesterol, and triglyceride levels in the experimental birds, while without adversely affecting their health or blood antioxidant status (p < 0.05); Utilizing various types of amaranths in the diets of laying hens did not result in any detrimental impacts on the physicochemical characteristics of eggs; Instead, it resulted in the production of eggs with decreased levels of yolk cholesterol and triglyceride; The omega-6 content in eggs and the ratio of omega-6 to omega-3 increased significantly (p < 0.05)	Kianfar et al. (2023)
56-weeks old Hy-line W-36 white leghorn laying hens	Iran	Amaranth grain	100−400 g/kg	The AG level had no significant effect on feed intake (FI) and percentage of hen day production (HDP); Nevertheless, it resulted in a significant decrease in both egg mass (EM) and egg weight (EW) (p < 0.01), as well as a notable deterioration in the feed conversion ratio (FCR) (p < 0.01); The inclusion of enzymes resulted in a substantial increase in EM (feed efficiency), EW (egg weight), and FCR (feed conversion ratio) at a statistically significant level (p < 0.01); The hens that were given a meal containing 200 g/kg AG with the addition of enzymes had the highest EM value and the lowest FCR value	Hosseintabar-Ghasemabad et al. (2022)
240-day-old broiler chickens (ROSS 308)	Czech Republic	Amaranth grain	7 %	The control group had a significantly higher carcass yield compared to the experimental group (p < 0.05); The inclusion of amaranth in the experimental group's rations did not affect protein concentrations compared to the control group; Glucose levels in the experimental groups of chickens were significantly lower (p < 0.05; p < 0.01)	Roučková et al. (2004)
32-weeks old laying hens (ISA SHAVER Line)	Poland	Amaranth grain	2, 5, and 10 %	The LDL cholesterol fraction experienced a significant decrease of approximately 45.6 % and 52.3 % in groups that were supplemented with 5 % and 10 % amaranth grain, respectively; The activity of alanine aminotransferase (ALT) was higher in groups supplemented with 2 % and 10 % amaranth grain compared to the control group	Króliczewska et al. (2008)
90-day old female broiler (Big Ray)	Italy	Amaranth grain	50, and 100 g/kg	The broilers that were fed diets supplemented with amaranth grain (AMG) had significantly lower growth performance (p < 0.05); In contrast, the broilers that were given a diet without AMG supplementation had higher levels of serum lipid peroxidation and lower levels of serum antioxidant power; Specifically, the broilers fed on a diet containing 100 or 50 g/kg AMG had significantly higher serum antioxidant power and lower serum lipid peroxidation levels, respectively, compared to the broilers without AMG supplementation; Additionally, the broilers fed on AMG diets had significantly lower levels of cholesterol and triglycerides (p < 0.05) compared to those given a diet without supplementation	Longato et al. (2017b)
20-week-old Hy-Line brown hens	Korea	Buckwheat grain	16 g/kg	The group that was treated with fermented buckwheat extract had increased levels of L-carnitine (13.6 %) and GABA (8.4 %) in the yolk; The difference in L-carnitine levels was statistically significant (p < 0.05); There was a significant increase (p < 0.05) in egg production (9.4 %), albumen weight (2.1 %), and shell weight (5.8 %); There was no statistically significant variation in yolk weight, total cholesterol (1.9 %), and triglyceride (4.9 %) levels. However, the yolk's triglyceride levels were reduced (p < 0.05).	Park et al. (2017)
One-day-old male Cornish-cross chicks	United States of America	Buckwheat grain	20,40, and 60 %	The findings revealed that incorporating up to 60 % buckwheat in broiler diets does not have a noteworthy impact on body weight gain. Nevertheless, at the 60 % inclusion level, there is a considerable decrease in feed efficiency	Jacob and Carter (2008)
29-day-old broiler chickens (ROSS 308)	Japan	Buckwheat grain	10 %	The retention of nitrogen in broilers reduced significantly (p < 0.05) when they were fed the negative control (NC) diet; However, this loss was reversed when buckwheat grain was added to their diet; The birds that were fed buckwheat grain showed a significant increase (p < 0.05) in the retention of total phosphorous (P) compared to those on the NC diet	Chowdhury et al. (2017)
74-wwek-old hens (ISA-Brown)	Italy	Buckwheat bran	30 %	The findings indicated a significant increase in egg production rate (p < 0.05) and feed consumption (p < 0.05) when incorporating buckwheat bran partially into the diet	Benvenuti et al. (2012)

### 7.1 Chicken

A study was performed to evaluate the effects of supplementing raw amaranth (*Amaranthus hybridus chlorostachys)* grain (RAG) to the diet of laying hens, either alone or combined with an enzyme blend. The study aimed to analyze the hens' productivity, blood biochemistry, and antioxidant status (Janmohammadi et al., 2023). The results suggested that feed with RAG levels exceeding 10%, significantly reduces the blood total cholesterol (TC). However, this also led to a detrimental effect on the feed conversion ratio (FCR) (p < 0.05), as evidenced by decrease in feed intake (FI), egg mass (EM), egg weight (EW), and hen daily production (HDP). As a result, the experimental group had lower overall production compared to the control group. In contrast, the inclusion of the enzyme blend resulted in a significant enhancement of the examined production characteristics (p < 0.05), except for HDP. The enzyme blend also effectively restored the performance efficiency when combined with small amount of RAG (10 %) (p < 0.05), and the groups that received both RAG and the enzyme blend exhibiting the lowest TC levels (p < 0.05). Janmohammadi et al. (2022) carried out two studies to determine the apparent metabolizable energy (AMEn) content of heat-treated (HTAG) and untreated (AG) amaranth grain in Ross-308 male broiler chicks, accounting for zero nitrogen balance. The study involved two enzyme levels (0 and 0.55 g/kg) and five different amaranth substitution rates (0, 150, 300, 450, and 600 g/kg). Metabolism experiments were conducted using the complete excreta collection method. The results indicated that the AMEn contents of HTAG were 3973 kcal/kg with enzyme addition and 3828 kcal/kg without enzyme addition. Enzyme supplementation resulted in 0.28 % increase in the energy content of UAG and a 3.8% increase in HTAG. AMEn value of HTAG exceeded UAG by 708 kcal/kg in enzyme-supplemented diets and by 573 kcal/kg in non-enzyme diets. The study found that heat treatment yielded more benefits compared to enzyme addition. However, the combination of heat treatment and enzyme insertion has a synergistic influence. were combined, resulting in an increased metabolizable energy content of amaranth in broiler diets. Alizadeh-Ghamsari et al. (2021) reported that feeding of 2 % of amaranth grain showed the higher body weight, average daily gain (ADG) and European broiler index (EBI) in Ross 308 mail broilers compared other treatment (0 %, 4 % and 6 %) (p < 0.05). Addition of 8 % of amaranth seeds in the feeding mixture of Ross 308 chickens decreased the body weight gains (p < 0.05) and increased the feed conversion ratio (Orczewska-Dudek et al., 2018). However, the addition of amaranth seeds in feed mixture increased the breast muscles. Popiela et al. (2013) assessed the impact of including extruded amaranth grains (AMG) into the diets of laying hens on their performance, egg qualities, composition of egg yolk fatty acids, and some blood parameters. A cohort of 60 Lohmann Brown laying hens, aged 24 weeks, was randomly divided into three dietary groups and fed for 10 weeks. The control group and two-treatment groups were supplemented with AMG at concentrations of 0%, 5%, and 10%. AMG supplementation led to increase in body weight (1.64, 1.66, and 1.72 kg for diets containing 0 %, 5 %, and 10% AMG, respectively (p < 0.01). The lowest feed conversion ratio and the highest egg production were observed in group supplemented with 5 % AMG. No significant variations were noted in egg shells weight, egg weight, albumin and yolk weight, Haugh units, thick albumen height and eggshell strength among hens fed with different AMG diets. Additionally, sensory analysis also found no discernible differences in eggs between the dietary groups. The fatty acid composition of egg yolk remained consistent across all treatments. However, in hens fed with 5 % AMG showed slightly higher level of polyunsaturated fatty acids (specifically linoleic acid), leading to a lower ratio of n-6 to n-3 acids. The study found that including AMG into the hens' diet at a 5 % level improved their performance without any adverse effects on their health or the quality and sensory characteristics of the eggs. Sayed et al. (2015) found that Cobb 500 broilers fed with buckwheat (0 %, 10 %, 20 % and 30 %) had no effect on growth and feed intake. Using larger concentrations of buckwheat (20 % or more) by itself, or lower concentrations of buckwheat (10 %) mixed with small quantities of chitosan, had a positive effect on the lipid profiles of the birds.

### 7.2 Other animals

Niewiadomski et al. (2016) assessed the utilization of amaranth meal as a dietary component for rainbow trout *Oncorhynchus mykiss*. Two experimental meals were generated, with one of them comprising 5 % amaranth meal and the other having 10 % amaranth meal. These feeds were then in comparison to a reference diet (commercial feed) that had equivalent amounts of specific nutrients. The feed was provided at a ratio ranging from 0.50 % to 1.71 % of the fish biomass for each individual group during a period of 21 days. Statistically significant differences (p < 0.05) were seen in the digestibility of crude protein and nitrogen free extract between the experimental groups and the reference group. The results demonstrated the viability of including amaranth flour as a component in animal feed. Poczyczyński et al. (2014), the researchers examined how substituting fish oil with amaranth oil in the diet of rainbow trout *Oncorhynchus mykiss* affected their body composition. Two experimental diets were used, one containing a concentration of 7.2 % amaranth oil and the other containing a concentration of 5 % amaranth oil. Each treatment was duplicated three times, with a sample size of 50 for each replication. The fish that were fed with feed containing the greatest quantity of amaranth oil had the highest specific growth rate, which was measured at 3.75 % per day. The fish meat in this group exhibited the most elevated concentrations of crude protein and crude fat, constituting 16.3 % and 10.5 % of body weight, correspondingly. The fatty acid composition of fish fat remained constant, but, the addition of more amaranth oil in the diet resulted in a significant decrease in eicosapentaenoic acid (C20:5n-3, EPA) and docosahexaenoic acid (C22:6n-3, DHA) in the fish fat. Stoldt et al. (2016) verified that the inclusion of buckwheat grains in the diet of dairy cows did not result in an increase in plasma quercetin levels. Furthermore, it had no impact on methane generation or milk yield. Jakubowska et al. (2013) utilized amaranth seeds as a dietary constituent for the purpose of feeding slaughter fowl. The experimental groups utilized feed containing 4 % and 7 % proportions of seeds from this plant. The scientists observed no discernible impact of seed application and level on the chemical composition and morphological characteristics of the pectoral and leg muscles. The application of amaranth seed and its level in the feed did not have any impact on the fatty acid profile, as compared to the control group. During the sensory evaluation, the pectoral muscles of quail that were fed with 4 % amaranth showed superior tenderness compared to the group that received 7 % amaranth and the control group. Zralý et al. (2004) performed a research investigation to analyze the impact of three meals, each containing 10 % amaranth, on several metabolic and health indicators, as well as growth efficiency in pigs. These diets were compared to a control diet that contained animal protein. Pigs weighing 18 kg the participants were allocated into two groups: the experimental group and the control group. The experimental groups were provided with meals consisting of either dried surface amaranth biomass or amaranth grain. These diets were either non-heat-treated or heat-treated through popping. The control group was given a diet including meat-and-bone meal. This feeding regimen lasted for 100 days. There were no notable disparities in the increases in live body weight seen between the experimental and control groups. The animals that were given the mixture comprising heat-treated amaranth had the largest daily body weight gain, which was recorded at 0.78 kg. On the other hand, the group that was fed non-heat-treated grain had the best feed conversion, which was measured at 2.45 kg.

## 8 Conclusion and future prospects

The extensive comprehensive exploration of amaranth and buckwheat in various facets of research, including their potential as human food sources, animal feed enrichment, and their impact on health parameters, presents a multifaceted understanding of their applications. For instance, the exploration of the glycaemic indices of snacks enriched with amaranth and the effect of rapeseed oil and amaranth on atherosclerosis-related parameters highlights the potential health benefits of incorporating these grains into the diet. Moreover, clinical studies evaluating the long-term health outcomes of regular consumption of these grain-based foods could provide valuable insights into their preventive and therapeutic roles in chronic disease management. The animal studies have also highlighted the potential of amaranth and buckwheat in enhancing animal feed formulations. Studies demonstrate improvements in productive performance, antioxidant status, and lipid profiles in chickens fed diets enriched with these grains. Additionally, the findings suggest that dietary supplementation with amaranth and buckwheat may contribute to weight management and overall health in animals. The potential of these grains extends beyond traditional human and animal consumption. In aquaculture, functional feed formulations incorporating plant products offer promise for sustainable growth. Studies examining the effects of amaranth meal and oil in fish feed reveal improvements in digestibility, growth rates, and body composition, indicating the feasibility of utilizing these grains in aquafeed formulation. Future research may explore the isolation, purification, and characterization of bioactive compounds from these grains, as well as their mechanisms of action and potential therapeutic applications. Furthermore, innovative delivery systems, such as encapsulation and nano-formulation, could enhance the bioavailability and efficacy of these compounds for targeted health outcomes. This review recommended that government and agriculture universities/institutions promote the cultivation of such crops by the farmers who are having small agriculture land with better productivity and for their better livelihood.

## CRediT authorship contribution statement

**Harsh Kumar:** Writing – original draft. **Shivani Guleria:** Writing – review & editing. **Neetika Kimta:** Conceptualization, Writing – review & editing. **Rajni Dhalaria:** Conceptualization, Writing – review & editing. **Eugenie Nepovimova:** Resources. **Daljeet Singh Dhanjal:** Writing – review & editing. **Suliman Y. Alomar:** Resources. **Kamil Kuca:** Conceptualization, Formal analysis, Resources, Supervision.

## Declaration of competing interest

The authors declare that they have no known competing financial interests or personal relationships that could have appeared to influence the work reported in this paper.

## Data Availability

Data will be made available on request.
